# Early Clinical Outcomes (ECO) in the Era of Rapid Recovery Total Joint Arthroplasty: A Comparative Evaluation of Three Bearing Surfaces

**DOI:** 10.7759/cureus.78398

**Published:** 2025-02-02

**Authors:** Jared Hammond, Benjamin Huang, Russell T Nevins

**Affiliations:** 1 Medicine, Arkansas College of Osteopathic Medicine, Fort Smith, USA; 2 College of Osteopathic Medicine, Touro University Nevada, Henderson, USA; 3 Orthopaedics, Valley Health System, Las Vegas, USA

**Keywords:** early postoperative pain, medial pivot knee, opioid utilization, outpatient surgery, pain reduction, posterior stabilized knee, primary total knee arthroplasty, retrospective chart review, rotating platform cruciate retaining knee

## Abstract

Background and objective

Outpatient total knee arthroplasties (TKAs) for low-risk patients are growing in popularity, thanks to expedited recovery and reduced hospital stays. Early postoperative pain reduction has been associated with improved long-term functional outcomes. This study aimed to compare three knee implant systems - posterior-stabilized, medial-pivot, and rotating platform cruciate-retaining (RPCRF) - in terms of their impact on early postoperative pain and opioid use.

Methods

A retrospective analysis was conducted, involving 94 posterior-stabilized TKAs, 104 medial-pivot TKAs, and 101 RPCRF TKAs by a single surgeon at two hospitals from June 2020 to March 2022. Pain levels per the Numerical Rating System were obtained from the patients' preoperative and postoperative visits at two weeks, six weeks, and 12 weeks. The daily milligram morphine equivalents (MME) in opioid-naive patients were calculated and compared between the three systems. The Kruskal-Wallis and Dunn tests were employed for all calculations to obtain p-values.

Results

Posterior-stabilized knees had a significantly larger reduction in pain levels compared to medial-pivot and RPCRF systems at two and six weeks postoperatively. There were no significant differences at the 12-week assessment. The daily MMEs prescribed were similarly reduced with posterior-stabilized knees at two and six weeks, with no difference at 12 weeks.

Conclusions

Our findings suggest that posterior-stabilized knee implants may offer superior short-term pain reduction and reduced opioid use requirements after outpatient TKA. Further research with randomized and blinded prospective trials is needed to elucidate improvements in patient satisfaction and functional outcomes.

## Introduction

Total knee arthroplasties (TKAs) are vital in reducing patients’ pain from osteoarthritis. Of note, 3.48 million TKAs are performed annually; these are one of the most common inpatient surgical procedures in the United States with a growing number of TKAs performed in the outpatient setting [1,2]. Long-term outcomes are rightfully a major research focus as revision arthroplasty significantly reduces the quality of life [3,4,5]. Short-term complications and pain have been associated with lower satisfaction in patients receiving total hip arthroplasty [6]. Lakra et al. and Lo et al. found that early postoperative pain after TKA predicted worse long-term functional outcomes [7,8]. Superior functional outcomes are associated with less opioid consumption two weeks postoperatively [9,10]. Implant choice could affect the level of pain reduction and, as TKA becomes more routine, design variations may have to be considered for specific patient populations.

Notably, outpatient TKAs are driven by a focus on early rehabilitation and reduction in the duration of hospitalization [11]. Low-risk patients having outpatient total joint arthroplasty had improved long-term outcomes when rehabilitation was started earlier [12,13]. Also, patients receiving outpatient total hip arthroplasty had similar outcomes when compared to inpatients [14,15]. Optimized patient recovery in outpatient TKA could be the underlying reason for significant cost savings in outpatient surgery compared to inpatients [16,17]. Given this emphasis on the early postoperative period, there is scarce research examining the differences in TKA implant design on Early Clinical Outcomes (ECO). Samy et al. found no significant difference in range of motion between medial-pivot and posterior-stabilized knees [18]. Another study found that posterior-stabilized knees had a statistically significant difference in range of motion of 5° at six weeks, which persisted through the two-year assessment [19]. However, no studies have examined ECO using pain scores or opioid use. This retrospective study aims to analyze ECO based on a comparative analysis involving three knee implant systems to further optimize outpatient TKA outcomes.

## Materials and methods

This study involved a retrospective chart review analyzing three different knee systems by a single surgeon at two hospitals between July 2020 and March 2022. The designs are posterior-stabilized knees (LinkSymphoknee, Waldemar Link, Hamburg, Germany), medial-pivot (EvolutionMP, Microport Orthopedics, Arlington, TN), and a rotating platform with a cruciate retaining femur (RPCRF) (PFC Sigma RP, JNJ/DePuy, Raynham, MA). Ninety-four patients received a posterior-stabilized TKA, 104 patients received a medial-pivot TKA, and 101 patients received a RPCRF TKA. There were no major variations between surgical procedures. The procedures were performed using a tourniquet placed around the upper thigh, and a median parapatellar approach. Patellar resurfacing was performed in all of the TKAs.

This retrospective chart review was conducted with strict adherence to ethical guidelines and patient privacy regulations. All patient information was de-identified. The Institutional Review Board (IRB) waived the requirement for informed consent as the data were extracted from routine medical care. This study did not receive any funding.

Implants

Posterior-Stabilized (LinkSymphoknee, Waldemar Link)

This posterior-stabilized implant replaces the role of the posterior cruciate ligament (PCL) by using a polyethylene post and femoral cam. The combination of the two creates a physiological similarity to the PCL, preventing anterior translation of the femur on the tibia and allowing femoral rollback during knee flexion.

Medial-Pivot (EvolutionMP, Microport Orthopedics)

The medial-pivot knee system attempts to reproduce the physiological kinematics of the knee. A ball and socket type movement within a concave constrained medial polyethylene acts as a pivot center. The lateral flatter polyethylene surface allows the lateral femoral condyle to rotate posteriorly during flexion. These motions combined in the lateral and medial compartments theoretically reproduce the physiological knee biomechanics

Rotating Platform Cruciate-Retaining (PFC Sigma RP, JNJ/DePuy)

This system combines a medial and lateral high surface area: a highly congruent tibial articular surface with the polyethylene allowed to rotate upon a center keel in the tibial base plate. This type of prosthesis may help replicate the small amount of rotation that a normal knee experiences when becoming fully extended and theoretically changes multidirectional wear to unidirectional wear.

Data collection

After obtaining IRB approval, patient data were gathered from electronic medical records. This data consisted of age, weight, BMI, previous medical conditions such as heart disease, hypertension, diabetes, cancer, and smoking status. Pain levels were also collected from patient medical records, which used the Numerical Rating System (NRS) from the preoperative visit, as well as two weeks postoperatively, six weeks postoperatively, and three months postoperatively. The differences between the pain levels from the preoperative visit and each of the postoperative visits were calculated. These calculations show the change, or delta, in pain levels since the patient’s preoperative status. The following time intervals were analyzed: preoperative versus two weeks postoperative, preoperative versus six weeks postoperative, and preoperative versus three months postoperative.

A prescription monitoring program was used to assess opioid prescription patterns at two, six, and 12 weeks, as well as six months postoperatively. The type and dose of the opioids were accounted for and converted to daily milligrams of morphine equivalents (MME) to compare all opioids equally. Data on other analgesic medications were not collected.

Statistical analysis

The Kruskal-Wallis test (K-W), a nonparametric approach to the one-way ANOVA test, was used to identify significant differences between the delta of pain levels from the three systems at each time interval. The K-W test was used to determine the significance of the differences in daily MME at each postoperative time interval between the three systems. After the K-W test, Dunn’s test was used. Dunn's test was used as a post hoc test to determine the significance between pairs of data: posterior-stabilized versus medial-pivot and posterior-stabilized versus RPCRF knee systems. Dunn's test was used to compare the differences in pain levels and daily MME at each time interval between those pairs.

## Results

The records of 299 patients who underwent a TKA using either of the three systems were examined. Of them, 94 received a posterior-stabilized knee system, 104 medial-pivot, and 101 RPCRF. The demographic data of the groups are shown in Table 1. All patients from each group attended their two-week postoperative visit. Six weeks postoperatively, six posterior-stabilized patients were lost to follow-up while none from medial-pivot and RPCRF were lost. At three months, posterior-stabilized lost 12 patients, medial-pivot 10, and RPCRF 13 to follow-up.

**Table 1:** Demographic data of the patients SD: standard deviation

Variables	Posterior stabilized	Medial-pivot	Rotating platform cruciate retaining
Gender, n (%)			
Male	42 (44.7)	42 (40.4)	50 (49.5)
Female	52 (55.3)	62 (59.6)	51 (50.5)
Weight, pounds, mean ± SD	205.3 ± 48.6	196.7 ± 44.0	217.4 ± 43.8
BMI, kg/m^2^, mean ± SD	31.7 ± 6.0	31.8 ± 6.5	33.4 ± 5.3
Age, years, mean ± SD	67.3 ± 9.7	64.1 ± 8.9	68.4 ± 10.4
Health history, n (%)			
Diagnosis			
Osteoarthritis	94 (100)	104 (100)	101 (100)
Rheumatoid arthritis	8 (8.5)	11 (10.6)	11 (10.9)
Arthrofibrosis	1 (1.1)	4 (3.8)	3 (3.0)
Avascular necrosis	4 (4.3)	4 (2.9)	0 (0.0)
Heart disease, n (%)			
Diagnosed	23 (24.5)	40 (38.5)	30 (27.7)
Hypertension, n (%)			
Diagnosed	35 (37.2)	58 (55.8)	47 (46.5)
Smoking status, n (%)			
Current	11 (11.7)	10 (9.6)	20 (19.8)
Former	26 (27.7)	35 (33.7)	16 (15.8)
Non-smoker	57 (60.6)	59 (56.7)	65 (64.4)
Cancer, n (%)	27 (28.7)	42 (40.4)	24 (23.8)
History of cancer	27 (28.7)	42 (40.4)	24 (23.8)
Diabetes, n (%)			
Diagnosed	30 (31.9)	42 (40.4)	58 (42.6)

Average preoperative pain scores on the scale were 6.66 for posterior-stabilized knees, 6.17 for medial-pivot knees, and 5.55 for RPCRF knees. Between the preoperative and the two-week postoperative assessments, the posterior-stabilized knee system reduced pain by 1.52 points, medial-pivot by 0.52 points, and RPCRF by 0.40 points. 

**Figure 1:**
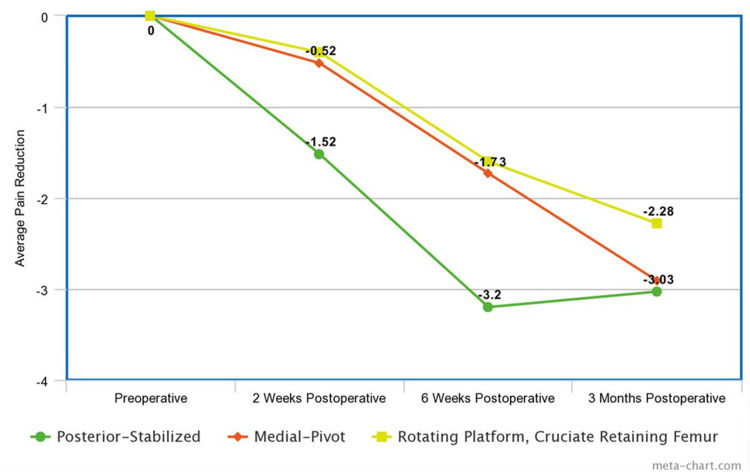
Absolute pain reduction at each postoperative visit

**Figure 2:**
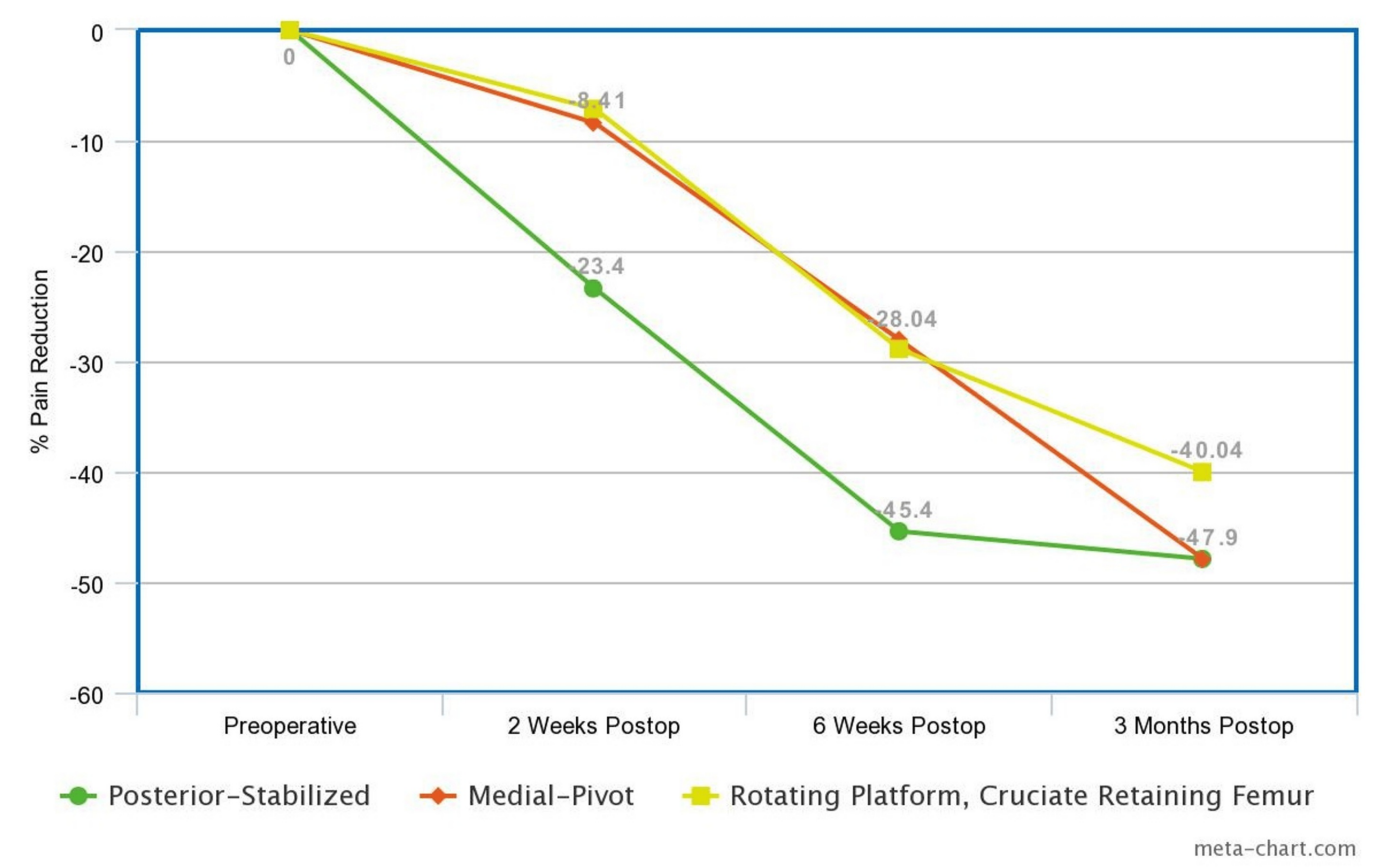
Relative pain reduction at each postoperative visit

A K-W test was run, yielding an H-statistic of 8.96 and a p-value of 0.0114. Dunn’s test was run to compare the posterior-stabilized knee to the others, giving a p-value of 0.018 between posterior-stabilized and medial-pivot and a p-value of 0.005 between posterior-stabilized and RPCRF. Between the preoperative and six-week postoperative assessments, a mean reduction in pain score of 3.20 was found in the posterior-stabilized, 1.73 in the medial-pivot group, and 1.60 in the RPCRF group. The K-W test was run, giving an H-statistic of 12.19 and a p-value of 0.002. Dunn’s test revealed a p-value of 0.006 between posterior-stabilized and medial-pivot. Between posterior-stabilized and RPCRF, Dunn’s test gave a p-value of 0.001. From preoperative to three months postoperative, the posterior-stabilized knee’s delta was 3.03, while that of medial-pivot was 2.91, and RPCRF 2.28. The K-W test gave an H-statistic of 2.51 and a p-value of 0.285. Dunn's test gave a p-value of 0.913 between posterior-stabilized and medial-pivot and 0.331 between posterior-stabilized and RPCRF. Absolute and relative pain reduction are shown in Figures 1-2.

The normalized MMEs were compared across patients taking oxycodone, hydrocodone, and morphine postoperatively as well as across each group. Preoperative opioid use was an exclusion factor, resulting in 77 posterior-stabilized patients, 89 medial-pivot patients, and 75 RPCRF patients being included in this analysis. All patients were prescribed opioids immediately after surgery according to the opioid protocol. Other analgesia was not accounted for in this MME calculation.

**Figure 3:**
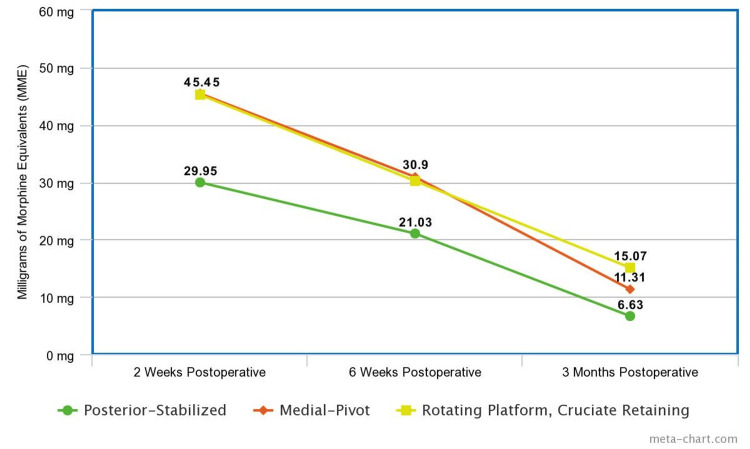
Average daily milligrams of morphine equivalents (MME) at each postoperative visit

When calculating the average daily MME, only those patients who were opioid-naive before surgery were included. This resulted in the inclusion of 77 patients who received a posterior-stabilized knee, 89 medial-pivot, and 75 RPCRF. At the two-week postoperative assessment, patients who received a posterior-stabilized knee had an average daily MME prescribed of 29.59, medial-pivot 45.45, and RPCRF 45.24. The K-W test gave an H-statistic of 30.07 and a p-value <0.001. Dunn's test showed a p-value of <0.001 between both pairs of posterior-stabilized and medial-pivot and posterior-stabilized and RPCRF. At six weeks postoperatively, the average daily MME prescribed for the posterior-stabilized group was 21.03, while it was 30.90 for medial-pivot and 30.20 for RPCRF. The K-W test gave an H-statistic of 6.48 and a p-value of 0.039. Dunn’s test between posterior-stabilized and medial-pivot knees gave a p-value of 0.028 and between posterior-stabilized and RPCRF, 0.025. The average daily MMEs at each postoperative visit are depicted in Figure 3.

## Discussion

This retrospective analysis found significantly reduced early opioid use and superior change in NRS scores with a posterior-stabilized knee implant compared to RPCRF and medial-pivot knees. With growing emphasis on early recovery and reduced hospitalization durations, a posterior-stabilized implant design could improve ECO and ultimately improve patient satisfaction with the prosthesis.

At the two-week and six-week postoperative assessments, patients who received the posterior-stabilized implant had a significantly greater reduction in self-reported pain levels than medial-pivot and RPCRF implants (p=0.0114 and 0.002, respectively). At the two-week postoperative assessment, patients with posterior-stabilized implants reported significantly lower pain scores compared to those with medial-pivot or RPCRF implants. This difference was more than triple the reduction observed in the two other groups. At the six-week assessment, posterior-stabilized implants continued to show a significant advantage, reducing pain scores by approximately double compared to the other designs. By the three-month assessment, there was no significant difference in reported pain scores among the three implant types. Although these results do not suggest that long-term clinical outcomes could be improved through early pain reduction, early improvements in ECO through implant selection could be explored in future research measuring patient satisfaction with TKA.

Similar to pain scores, posterior-stabilized knees were prescribed significantly fewer MME than medial-pivot and RPCRF implants (p<0.001). At the six-week postoperative assessment, posterior-stabilized implants also showed a significant decrease in the amount of opioids prescribed compared to the two other implant designs (p=0.039). As early higher opioid use is associated with worse outcomes, this early reduction in prescribed MMEs with posterior-stabilized knee implants warrants further evaluation.

As the literature and the data in this study suggest that functional outcome measures longer than 12 weeks status post-TKA are identical, the level of pain reduction and concomitant reduction of opioid use can benefit patient satisfaction with TKA [20-22]. Posterior-stabilized knee implant designs appear to provide early benefits thanks to improved stability. The cam-post articulation is believed to bear valgus/varus stress in flexion, especially in a deep squat motion that is associated with the ability to participate in functional activities [23,24]. This improved stability compared to cruciate-retaining and RPCRF TKA could increase early activity and explain this study's findings. While there is no consensus on any implant design having distinctly superior long-term outcomes, proponents believe that posterior-stabilized knee implants have greater stability in exchange for reduced flexion [25]. Given the emphasis on rapid recovery and early discharge in TKA, posterior-stabilized knees could be preferred for TKAs done in an outpatient setting. 

Limitations and future directions

This retrospective study did not involve matching, which could have confounded results due to expertise bias. There is also a risk of attrition bias although it is unlikely that there was a difference in patients lost to follow-up in the three knee systems. The primary outcomes measured were pain and opioid use, which are less comprehensive than patient-reported outcome measures. NRS score is a subjective measure of a patient’s pain, which is affected by recall bias. Opioid consumption was measured through a prescription monitoring program, which is limited by medication adherence, social determinants of health, and lack of patient-level data on opioid consumption. Specifically, data on other analgesic medications was not collected; opioid consumption and other analgesia in this study reduced pain scores and if this affected the outcomes of this study, opioid consumption would have been greater and correlated with lower pain scores. This relationship was not explored and future research should ensure this is not a confounding variable in prospective randomized trials. Subgroup analyses, economic evaluations, and correlation with long-term outcomes were also not included. Finally, this data originated from a single surgeon at two institutions, which could reduce external validity.

Future research could look at preoperative patient optimization in combination with implant design and postoperative rehabilitation, where the combination could further allow TKA to become a routine and standardized procedure. In today’s environment, the community standard considers all posterior-stabilized, cruciate retaining, and mobile-bearing implants as uniform. However, specific biomechanical changes between brands and companies may influence the outcome of TKA. The posterior-stabilized implant used in this study has a specific placement of the post in the AP direction and the angle of the post-cam mechanism is different from other systems. Further research should investigate if the findings of this study could be attributed to this. Finally, this study did not establish a link between ECO and long-term outcomes although current literature suggests a correlation. Future studies could randomize patients to posterior-stabilized implants vs. other designs in the outpatient setting and correlate ECO with long-term function and satisfaction with TKA. Patients with specific instability could benefit more from the stability offered by a posterior-stabilized knee design.

## Conclusions

Based on our findings, posterior-stabilized knee replacement significantly reduced patient pain levels and opioid use earlier in the postoperative period compared to medial-pivot and RPCRF knee systems. Hence, our results endorse the use of a posterior-stabilized implant design in TKA to achieve early pain reduction and reduced opioid use.
